# Comparative proteomics analysis of the mouse mini-gut organoid: insights into markers of gluten challenge from celiac disease intestinal biopsies

**DOI:** 10.3389/fmolb.2024.1446822

**Published:** 2024-08-28

**Authors:** Robert Moulder, Santosh D. Bhosale, Keijo Viiri, Riitta Lahesmaa

**Affiliations:** ^1^ Turku Bioscience Centre, University of Turku and Åbo Akademi University, Turku, Finland; ^2^ InFLAMES Research Flagship Center, University of Turku, Turku, Finland; ^3^ Celiac Disease Research Center, Faculty of Medicine and Health Technology, Tampere University and Tampere University Hospital, Tampere, Finland; ^4^ Institute of Biomedicine, University of Turku, Turku, Finland

**Keywords:** organoid, mini-gut, proteomics, celiac disease, gluten challenge

## Abstract

**Introduction:**

Organoid models enable three-dimensional representation of cellular systems, providing flexible and accessible research tools, and can highlight key biomolecules. Such models of the intestinal epithelium can provide significant knowledge for the study of celiac disease and provide an additional context for the nature of markers observed from patient biopsy data.

**Methods:**

Using LC–MS/MS, the proteomes of the crypt and enterocyte-like states of a mouse mini-gut organoid model were measured. The data were further compared with published biopsy data by comparing the changes induced by gluten challenge after a gluten-free diet.

**Results and discussion:**

These analyses identified 4,850 protein groups and revealed how 400 putative biomarkers of dietary challenge were differentially expressed in the organoid model. In addition to the extensive changes within the differentiated cells, the data reiterated the disruption of the crypt–villus axis after gluten challenge. The mass spectrometry data are available via ProteomeXchange with the identifier PXD025690.

## 1 Introduction

As a model for biomedical research, organoid systems can renew, differentiate, and organize into structures, enabling the three-dimensional representation of living systems *in vitro* (Sato et al., 2009). In particular, these provide opportunities to study disease processes and conduct drug screening, mimicking model organs such as the kidney, lung, gut, and brain (Clevers, 2016; Yin et al., 2019). Among these targeted organs, the intestinal epithelium of the gut represents one of the fastest proliferating mammalian tissues and is renewed every 4–5 days (Sato et al., 2009). As a resource for research, the so-called mini-gut model is important in the study of emerging autoimmune disorders such as celiac and Crohn’s disease, ulcerative colitis (Angus et al., 2020), and intestinal cancer.

The intestinal epithelium is composed of a single cell layer that creates a series of protruding villi and invagination luminal crypts, which are further distinguished by a range of different cell types (Sasaki et al., 2017). Stem cells in the crypt and progenitor cells in the transit amplifying zone proliferate, differentiate, and migrate, resulting in the expression of the ensemble of distinct cell types. Among these, enterocytes are the most abundant and are involved in nutrient absorption, while others include goblet, Paneth, and microfold cells, which secrete the mucus layer, produce antimicrobial peptides, and sample and present antigens, respectively.

One of the key manifestations of celiac disease (CD) is the impeded differentiation of the intestinal epithelium. Accordingly, identification of the molecules secreted by the epithelium in the blood could serve as proxy indicators of intestinal health. In this respect, analysis of the mini-gut organoid model can provide insights into the intestinal compartment and secreted epithelial cell-specific molecules while overcoming limitations imposed by extra-epithelial tissues or the malignant quality of cancer cell lines such as Caco-2 (Sun et al., 2008).

Mini-gut models of the intestine have been previously created from Lgr5+ intestinal epithelial stem cells grown with a balance of growth factors and inhibitors on Matrigel (Sato et al., 2009). As with other organoids, detailed biochemical analysis of the transcriptome and proteome enables recognition of changes in the system with different stimuli (Gonneaud et al., 2017). Lindeboom et al., for example, used an extensive multi-omics approach to provide a holistic view of the molecular mechanisms associated with intestinal organoid differentiation (Lindeboom et al., 2018).

**FIGURE 1:**
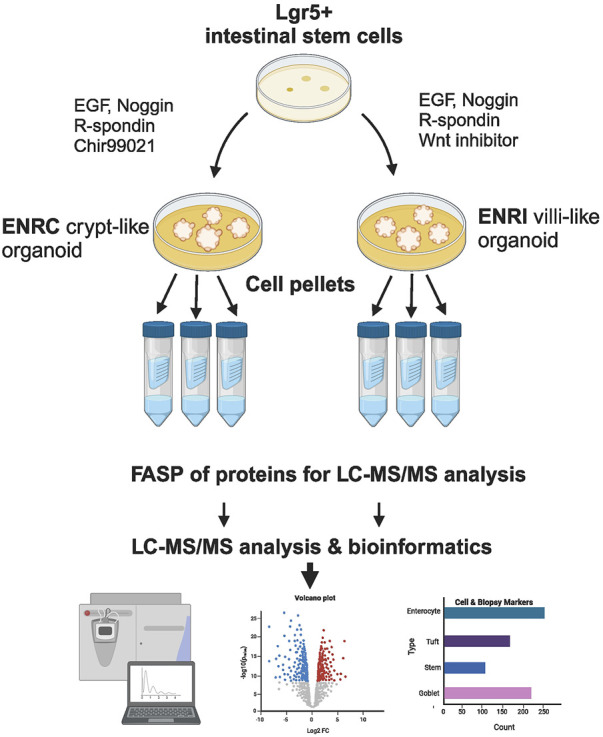
Schematic of the cell culture and preparation. Mouse Lgr5+ intestinal cells were grown in Matrigel™, and with the variation of stimulation vs inhibition of Wnt signaling, the resulting mini-gut organoids were maintained as crypt-like cells (ENRC) or differentiated enterocytes (ENRI), respectively. Following filter-assisted sample preparation (FASP), the cellular lysates from three replicates were analyzed by liquid chromatography and tandem mass spectrometry (LC–MS/MS). Subsequent statistics and bioinformatics were used to interpret the data, with comparisons made with markers reported from celiac disease biopsies. (Figure created with BioRender.com).

In the context of potential markers of celiac disease, we have conducted proteomics analysis of a mouse mini-gut model (Figure 1). In this study, combination of the measurement of the cellular proteomics of crypt and enterocyte-like organoid states and comparison of these with markers reported from human CD biopsies (Dotsenko et al., 2020; Stamnaes et al., 2021) revealed the overlap with proteins differentially expressed between the two conditions. The transposition of the expression of these markers in relation to the organoid model revealed further details of the nature of the disease-associated disruptions.

## 2 Methods

### 2.1 Cell culture

Mouse intestinal crypts, containing Lgr5+ intestinal stem cells, were isolated and maintained in a basal medium of advanced Dulbecco’s modified Eagle medium/F12 that included 100 U/mL pen/strep, 10 mM HEPES, 2 mM Glutamax, and 1 mM N-acetyl cysteine, in addition to N2 and B27 supplements. To establish the crypt cell-like condition, the basal medium was supplemented with 50 ng/mL mouse recombinant EGF, 100 ng/mL mouse recombinant Noggin, 1 μg/mL human recombinant R-spondin, and 10 µm glycogen synthase 3 beta antagonist (Chir99021), denoted as ENRC. For the culture of differentiated cells, the media supplementation differed with the reduction of human recombinant R-spondin (300 ng/mL) and the substitution of the Wnt inhibitor IWP-2 (5 µm) for Chir 99021, denoted as ENRI. The mini-guts were grown in Matrigel for 3 days (no media change) as triplicates divided into 12 wells per replicate (24-well plates), with 0.35 mL medium per well, i.e., 4.2 mL per replicate. Prior to their analysis, the cells (as 3 × 12 wells of either ENRC or ENRI) were collected, washed with PBS, and snap-frozen in liquid nitrogen.

### 2.2 Proteomics sample preparation

The cell samples were prepared with a filter-assisted sample preparation (FASP) protocol (Wisniewski et al., 2009). Briefly, the samples were denatured with a lysis buffer of 4% SDS, 0.1 M DTT in 0.1 M Tris-HCl at pH 7.6 by using a combination of heat and ultrasonication. The protein lysate (60 µg) was transferred into a centrifugal molecular weight filter (30 kDa MWCO, Millipore). Buffer exchange (x2) with a urea buffer (8 M urea in 0.1 M Tris-HCl pH 8.0) was used to displace SDS. The reduced cysteine residues were then alkylated using iodoacetamide (0.5 M, 20 min in darkness), followed by buffer exchange (x2) to remove the alkylating agent. After buffer exchange to the digestion buffer (0.1 M Tris-HCl pH 8), sequencing-grade modified trypsin was added (1:30), followed by overnight incubation at 37°C. The peptide digests were acidified and desalted using Sep-Pak C_18_ solid-phase extraction cartridges (Waters) (Moulder et al., 2016).

### 2.3 LC–MS/MS

The digests of the cell samples were analyzed by LC–MS/MS in triplicate with sample randomization. An Easy-nLC 1200 coupled to Q Exactive HF was used (Thermo Fisher Scientific). The separations were conducted by using a 75 μm × 400 mm analytical column, packed with 1.9 µm ReproSil C_18_ (Dr Maisch GmbH). A separation gradient from 7 to 25% B in 75 min, to 35% in 15 min, and to 100% in 10 min was used at a flow rate of 300 nL/min. MS/MS data were acquired in the positive ion mode with a data-dependent acquisition setting for higher-energy C-trap dissociation (HCD) of the 12 most intense ions (m/z 300–1700, charge states >1+, NCE = 27). MS1 spectra were acquired with the resolution set to 120,000 (at m/z 200), with a target value (AGC) of 3 × 10^6^ ions and a maximum injection time of 100 ms. MS/MS spectra were acquired in the Orbitrap with a resolution of 15,000 (at m/z 200), a target value of 5 × 10^4^ ions, a maximum injection time of 200 ms, and the lowest mass fixed at m/z 120. Dynamic exclusion was set to 30 s.

### 2.4 LC–MS/MS data processing and statistical analysis

The mass spectrometry raw files were searched against a UniProt FASTA sequence database of the mouse proteome (version June 2016, 17,042 entries) using MaxQuant software version 1.5.5.1 (Cox and Mann, 2008) with the Andromeda search algorithm (Cox et al., 2011). Trypsin digestion, with a maximum of two missed cleavages, carbamidomethylation of cysteine as a fixed modification, and variable modification of methionine oxidation and N-terminal acetylation were specified in the searches. A false discovery rate (FDR) of 0.01 at the peptide and protein levels was applied. Label-free quantification (MaxLFQ) was used with the match between run options enabled (Cox et al., 2014).

The search results were further preprocessed and filtered using Perseus software (Tyanova et al., 2016). Identifications from the reversed sequence search, non-mouse contaminants, and proteins only identified by variably modified peptides were removed. Protein LFQ values were log_2_-transformed, followed by filtering to retain proteins with two valid values in at least one group (ENRI, ENRC, or either media). Missing values were imputed as half the minimum intensity. Statistical analyses were carried out for the medians of the technical replicates for the imputed data using the reproducibility-optimized test statistic (ROTS) (Elo et al., 2009). A false-discovery rate of 0.05 was applied, and a two-fold cut-off was used to represent the largest differences.

### 2.5 Bioinformatics

To analyze the functional annotation for the proteins and visualize known interactions and pathways, the DAVID (Haung et al., 2007) and STRING (Szklarczyk et al., 2011) web-based applications were used. Further analyses were made with Venny (Oliveros, 2020), R scripts, and Ingenuity Pathway Analysis (QIAGEN).

### 2.6 Cross-comparison with published biopsy studies and other data

As a reference for the organoid model in relation to CD, comparisons were made against markers identified from human biopsy studies (Dotsenko et al., 2020; Stamnaes et al., 2021). First, from genome-wide 3’ RNA-seq analysis of PAXgene-fixed paraffin-embedded (PaxFPE) duodenal biopsies, collected from 15 CD patients, markers have been reported that reflect the influence of strict long-term gluten-free diet (GFD), prior to and post gluten challenge (PGC), and from six disease control (DC) patients (non-CD although biopsied for another reasons) (Dotsenko et al., 2020) and second, from proteomics analysis of formalin-fixed paraffin-embedded (FFPE) biopsies (n = 20) collected before and after a 14-day gluten challenge (Stamnaes et al., 2021). The latter included direct tissue analysis and laser capture microdissection (LCM) isolation of the epithelial cell compartment. To enable the comparison with the latter panels, the proteins and genes were mapped between the mouse and human analogs.

The proteins detected and quantified were further compared with protein expression data and other data sets concerning the mini-gut and organoids from human biopsies comparing CD and controls. These included the mini-gut data from Lindeboom et al. (2018), who similarly used label-free proteomics to analyze a mouse organoid model, the RNA-seq comparison of intestinal organoids from the duodenal biopsies of non-celiac (NC) and CD patients by Freire et al. (2019), and the comparison of differences in RNA between organoids from CD biopsies and healthy controls by Dieterich et al. (2020). In addition, reference was made to the proteomics database of Gebert and co-workers, who analyzed the proteomes of intestinal crypts from mice across different anatomical regions and ages (Gebert et al., 2020), and the marker gene lists of Haber et al. (2017) from the single-cell analysis of mouse small intestine and organoids. For the comparison, the accession numbers and/or genes reported were mapped to available UniProt identifiers. Reanalysis of the re-mapped, supplementary protein intensity data (Lindeboom et al., 2018) was carried out using the ROTS (Elo et al., 2009).

These mass spectrometry proteomics data have been deposited to the ProteomeXchange Consortium (http://proteomecentral.proteomexchange.org) via the PRIDE partner repository (Perez-Riverol et al., 2019) with the dataset identifier PXD023737.

### 2.7 Quantitative reverse transcription PCR (qRT-PCR)

To further substantiate the model, qRT-PCR analysis of the selected intestinal markers was carried out as previously described (Oittinen et al., 2017). RNA was extracted from cells/tissues using TRIzol (Invitrogen™, Thermo Fisher Scientific, Carlsbad, CA) according to the manufacturer’s protocol. In brief, cDNA was synthesized with an iScript cDNA synthesis kit, quantitative PCR reactions were performed with SsoFast™ EvaGreen Supermix, and reactions were run in CFX96 real-time PCR detection systems (Bio-Rad, Hercules, CA). In addition to the targets, Lgr5, Alpi, Lyz, Chga, and Muc2, GAPDH was measured for normalization.

The qRT-PCR analyses of these targets from 1 to 4 days of culture were used to define 3 days as the optimal time point. For the current data, the measurement was taken for one biological replicate with three technical replicates. The list of the qRT-PCR oligonucleotides used and data are shown in the Supplementary Material.

## 3 Results

**FIGURE 2:**
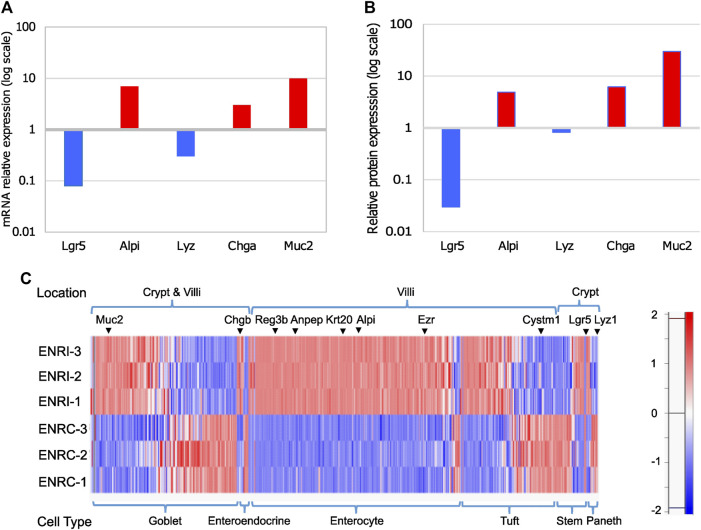
Comparison of intestinal cell markers in ENRI vs ENRC cells: **(A)** RT-qPCR analyses were carried out to distinguish the different intestinal cell types using the following markers: Lgr5 (leucine-rich repeat-containing G-protein-coupled receptor 5) for intestinal stem cells, Alpi (intestinal-type alkaline phosphatase) for enterocytes, Lyz1 (lysozyme) for Paneth cells, Chga (chromogranin-A) for enteroendocrine cells, and Muc2 (mucin) for goblet cells. **(B)** Relative intensities of the intestinal cell markers tested by RT-qPCR were cross-checked in the proteomics data. The values are the differences of the log_2_ (LFQ intensities). **(C)** Protein expression for detected intestinal cell markers and their expected location (crypt or villi). The marker list is as described by Haber et al. [26]. Examples of the markers are marked. The protein intensities are Z-score-normalized, as represented in the color scale.

Using mouse Lgr5+ intestinal cells grown in Matrigel with the variation of either stimulation (ENRC) or inhibition (ENRI) of Wnt signaling, mini-gut organoid cells were maintained as crypt-like cells or differentiated enterocytes, respectively (Sato et al., 2009). As a measure of the relative polarization of the cell states, RT-qPCR comparison of the mRNA expression of intestinal cell markers between the ENRI and ENRC cells indicated the formation of enterocytes, enteroendocrine cells, and goblet cells (Figure 2A). Similarly, a comparison of the relative abundance of these targets from the proteomics data further supported these differences (**2b).** Additional evaluation of the cellular types associated with the mini-gut model was made by comparing the proteomics data with other markers of enterocytes, tuft cells, enteroendocrine, and stem cells (Haber et al., 2017), as discussed below (**2c**).

### 3.1 Characteristics of the differentiated and undifferentiated mini-gut proteomes

Label-free quantitative proteomics analysis was used to compare relative protein abundances between the undifferentiated, crypt-like (ENRC) and differentiated, villus-like (ENRI) cells of the mouse Lgr5+ intestinal stem cell mini-gut model (Figure 1). Of the 4,850 proteins characterized by at least two unique peptides, 4,600 were used for quantitative analysis (after filtering for missing values). Statistical analysis of the LFQ protein intensities using the ROTS statistic (Elo et al., 2009) indicated changes in 74% of the detected proteomes (FDR ≤0.05). Applying a stricter criteria to highlight the most distinct changes, using a two-fold cut-off (FC, i.e., log2 value = 1.0) included changes in ∼25% of the detected proteomes (Supplementary Figure S1).

**FIGURE 3:**
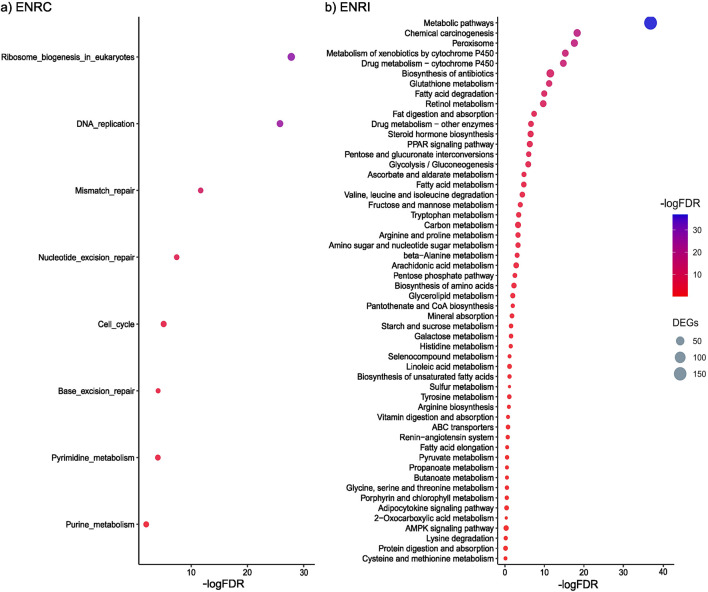
**(A)** KEGG pathways associated with the proteins differentially abundant in the ENRC cells. **(B)** KEGG pathways associated with the proteins differentially abundant in the ENRI cells. The inclusion criteria were a ROTS FDR ≤0.05 and a fold-change cut-off of 2. Comparison was made against the mouse proteome, and an FDR of 0.05 was applied.

Functional enrichment analysis of proteins more abundant in the ENRC cells revealed KEGG pathways associated with processes that maintain stem cell homeostasis, such as ribosome biosynthesis, mismatch repair, and nucleotide excision repair (Figure 3A). In particular, among the proteins common to these pathways were a series of DNA polymerases (i.e., Pole, Pold1, and Pold3) and replication factors (i.e., Rfc1–Rfc5), representing the machinery and accessory proteins for DNA synthesis and replication (Supplementary Table S1; Supplementary Figure S2).

With the functional enrichment analysis of proteins more abundant in the villi-like differentiated cells, a strong representation of KEGG pathways associated with food, drug, and chemical processing in the gut, such as the metabolism of starch and sucrose, galactose, carbon, fatty acid, retinol, and drugs, was observed (Figure 3B). For example, members of the cytochrome p450 family were specific to the differentiated cells (e.g., Cyp2c40, Cyp2d26, and Cyp3a11), as well as proteins from several solute carrier families, including roles in peptide, glucose, and ion transport (e.g., Slc6a20a, Slc2a5, and Slc6a20a).

There were 280 enterocyte markers detected based on the comparison with single-cell RNA-seq data previously reported from organoids and the mouse small intestine. In addition, there were 128 tuft cell, 16 enteroendocrine cell, 45 stem cell, and 15 Paneth cell markers (Figure 2C; Supplementary Table S2). In common with previous proteomic analysis of the mini-gut (Lindeboom et al., 2018), the latter include the enterocyte markers Vil1 (Sato et al., 2011), Alpi (Tetteh et al., 2016), Krt20, and Anpep (Merlos-Suárez et al., 2011), with the addition of the tuft cell marker Basp1 (Haber et al., 2017). A heat map representation of the Z-score normalized protein intensities is shown in Figure 2C for the detected markers, which is sorted by the anticipated location and relative abundance.

Clear examples of the differences between the organoids were illustrated when focusing on the proteins detected exclusively in the different states. For example, in keeping with their pluripotency, a group of proteins unique to the ENRC cells were associated with DNA replication, mitosis, and cytokinesis, e.g., Pole, Zwilch, and Kif20b (Supplementary Figure S3; Supplementary Table S3). Analysis of their common interactions further underlined this connectivity, linking the oncoprotein Cip2a (also unique to the ENRC cells), which is known to stabilize Myc, an important transcription factor in the maintenance of stem cell pluripotency. The forkhead box transcription factor Foxal was also unique to the ENRC cells. This has essential roles in organogenesis and tissue differentiation and is associated to cell differentiation in the intestine (Ye and Kaestner, 2009). The proteins exclusively detected in the ENRI cells (Supplementary Table S4) included solute carrier proteins involved in the transport of various ions, amino acids, sugars, and transferases, as indicated above. Similarly, among these, trehalase is a glycoside hydrolase produced on the brush border of the small intestine. Other differentially expressed brush border enzymes otherwise detected in the data include gamma-glutamyltranspeptidase 1 and intestinal-type alkaline phosphatase (Iap).

### 3.2 Comparison with putative CD markers

**FIGURE 4:**
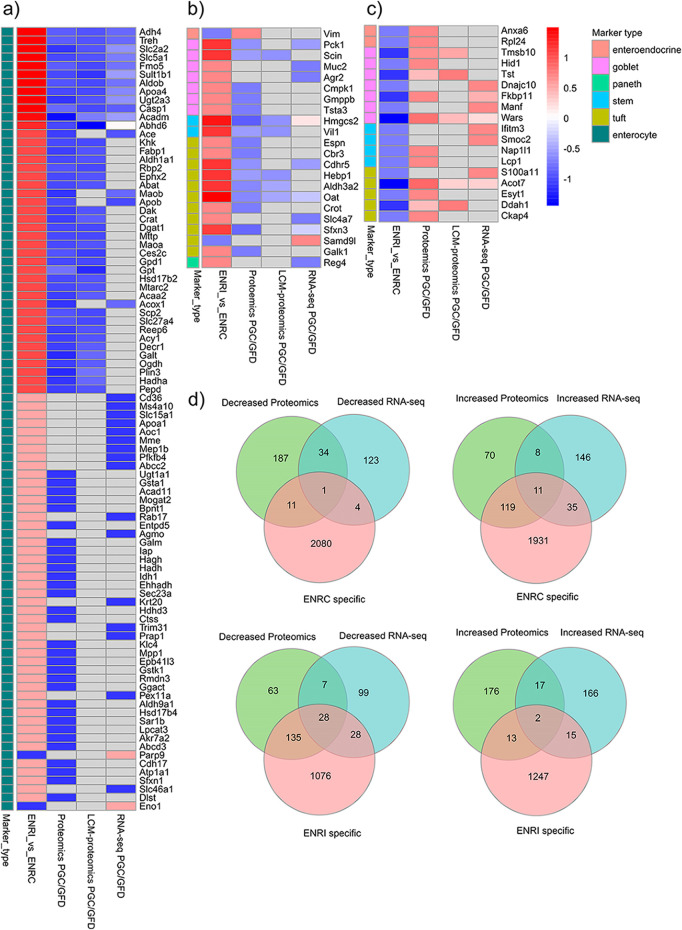
Cross-comparison of the genes and proteins detected as differentially expressed in the GC vs. GFD biopsies with intestinal cell markers and other proteins detected in the mini-gut organoid. **(A)** Enterocyte; **(B, C)** other intestinal cell markers. To highlight the larger differences, arbitrary expression change cut-offs were applied. For the biopsies, an absolute difference of log2 intensities of 0.6 was used, while for organoids, a cut-off of 1.0 was applied. The proteins shown in the figure represent the 146 markers that were within the applied cut-off values. The full lists of proteins are included in the ESM. **(D)** Comparison of all the differentially expressed proteins that were associated with either the organoid ENRI or ENRC cells and their analogs in the biopsy data sets. For this comparison, the mouse organoid proteins that were differentially abundant (ROTS FDR ≤0.05) were mapped to their human analogs.

To transpose the observations from these organoid measurements into the context of CD markers in humans, comparisons were made against targets identified from proteomics and RNA-seq analysis of CD biopsy samples (Dotsenko et al., 2020; Stamnaes et al., 2021). In addition to establishing common proteins, these comparisons provided a cross-reference of how the intestinal markers were affected and, thus, the nature of the cellular disruption (i.e., crypt or villi) from the CD/dietary challenge (Figures 4A–C). Relative to the list of markers reported from FFPE tissue biopsy proteomics analysis by Stamnaes et al. (2021), there were 178 ENRI-associated proteins and 143 ENRC-associated proteins, as assigned by their relative expression in the mini-gut analysis. On comparison with the RNA-seq data, analogs of 124 ENRI-associated and 51 ENRC-associated proteins were found (Figure 4D). Overall, the comparison reiterated how the markers detected in the biopsies presented an increased abundance of proteins reflecting the undifferentiated, ENRC-like state and a decrease in proteins associated with the differentiated, ENRI-like state. The detection of ENRC/crypt-like protein markers could, thereby, be a signature of the development of crypt hyperplasia. Regarding the protein and RNA-seq biopsy data sets, it was noted that the direction of change for matched genes and proteins was highly concordant.

Among the analogs of the organoid proteins more abundant in the crypt-like ENRC cells that were found at increased levels in the biopsy data, there were six members of the DNA replication licensing factor MCM complex (MCM2–6) (Table 1). Similarly characteristic of the ENRC cells and detected in the biopsy data were the nucleosome assembly protein 1-like 1 (Nap1l1) and Annexin A6 (Anxa6). Nap1l1 has been reported as a crypt stem cell marker and Anaxa6 as an enteroendocrine cell marker. Notably, with their comparison of organoids from NC and CD patients, Freire and coworkers observed the increase of several stem cell markers in the CD organoids (Freire et al., 2019). These included LGR5, the key marker of the mini-gut stem cells, and SMOC2, which is also predominant in the ENRC cells and was detected at increased levels in the RNA-seq analyses of Dotsenko et al. (2020). In the CD organoid comparison of Dieterich et al. (2020), genes involved in the epithelial-to-mesenchymal transition were decreased relative to the NC, which could account for the disruption of the crypt/villus axis in CD.

**TABLE 1:** Proteins associated with the ENRC cells and upregulated in the RNA-seq and proteomics biopsy comparisons. The negative ENRI–ENRC values reflect their low expression in the ENRI cells, i.e., expression in ENRC. In addition, the table includes three ENRI proteins that were upregulated in the biopsies. The table represents a subset of the larger differences, with the arbitrary expression change cut-offs of an absolute difference of log2 intensities of 0.6 for the biopsies and 1.0 for the organoid (all with FDRs <0.05).

Mouse gene	Cellular ENRI- ENRC (log2)	Biopsy tissue proteomics difference (log2)	Biopsy LCM proteomics difference (log2)	Biopsy RNA-seq difference (log2)	Protein name	Cell marker/protein type
Aurkb	−17.91	0.64	NA	NA	Aurora kinase B	-/kinase
Anxa1	−3.32	1.09	NA	NA	Annexin A1	-/enzyme
Impdh2	−2.26	0.87	0.62	NA	Inosine-5-monophosphate dehydrogenase 2	-/enzyme
Pcna	−2.01	0.73	NA	0.62	Proliferating cell nuclear antigen	-/enzyme
Ddx21	−1.89	1.19	0.66	NA	Nucleolar RNA helicase 2	-/enzyme
Nop2	−1.85	0.94	NA	NA	Probable 28S rRNA (cytosine-C (5))-methyltransferase	-/-
Anxa6	−1.79	0.97	NA	NA	Annexin A6	Enteroendocrine/ion channel
Mcm4	−1.61	1.28	0.90	0.53	DNA replication licensing factor MCM4	-/enzyme
Mcm3	−1.58	1.32	0.67	NA	DNA replication licensing factor MCM3	-/enzyme
Mcm6	−1.55	0.96	0.62	NA	DNA replication licensing factor MCM6	-/enzyme
Tmsb10	−1.53	0.90	0.74	NA	Thymosin beta-10	goblet/-
Mcm2	−1.49	1.68	1.68	NA	DNA replication licensing factor MCM2	-/enzyme
Nap1l1	−1.48	1.80	NA	NA	Nucleosome assembly protein 1-like 1	-/-
Mcm7	−1.46	1.59	0.71	NA	DNA replication licensing factor MCM7	-/enzyme
Mcm5	−1.45	1.37	0.67	0.81	DNA replication licensing factor MCM5	-/enzyme
Rbm3	−1.40	0.82	0.92	NA	RNA-binding protein 3	-/-
Hsph1	−1.19	0.88	0.53	0.57	Heat shock protein 105 kDa	-/-
Hid1	−1.16	0.71	NA	NA	Protein HID1	goblet/-
S100a11	−1.11	NA	NA	0.92	Protein S100-A11	tuft/-
Rcc2	−1.08	1.03	NA	NA	Protein RCC2	-/-
G3bp1	−1.07	0.96	NA	NA	Ras GTPase-activating protein-binding protein 1	-/enzyme
Tfrc	−1.07	1.11	0.92	0.54^a^	Transferrin receptor protein 1	-/transporter
Acot7	−1.04	1.12	0.59	0.56	Cytosolic acyl coenzyme A thioester hydrolase	-/enzyme
Pigr	2.20	1.00	5.93	0.72	Polymeric immunoglobulin receptor	/transporter
Vim	1.15	1.17	NA	NA	Vimentin	Enteroendocrine/-
Ctss	1.97	1.56	NA	NA	Cathepsin S	enterocyte/peptidase

a Indicates differences detected from the GFD vs. disease control (DC) comparison for the RNA-seq data (all others are gluten-free diet (GFD) vs. gluten challenge).

Included with the proteins/genes that were at lower levels in the biopsy data sets, there were 191 analogous organoid proteins that were associated with the ENRI cells, which included 58 enterocyte markers (Figure 4A). Examples of these are angiotensin-converting enzyme (Ace), trehalase, and phosphoenolpyruvate carboxykinase (Pck1, a goblet cell maker). These and other prominent examples are considered below, included in Tables 1–3. Here again, the overlap of the detected markers and the intestinal cell classifications are indicative of how CD and crypt hyperplasia result in a reduction of the mature cells of the villus.

**TABLE 2:** Proteins associated with the ENRI cells and downregulated in the RNA-seq and proteomics biopsy comparisons. All figures are differences of log2 values.

Mouse gene	Cellular ENRI- ENRC (log2)	Biopsy-proteomics (log2)	Biopsy-LCM proteomics (log2)	RNA-seq difference (log2)	Protein name	Marker and protein type
Adh4	20.38	−1.27	−1.4	−0.71	Alcohol dehydrogenase 4	Enterocyte/enzyme
Treh	19.79	−1.35	−1.65	−0.55	Trehalase	Enterocyte/enzyme
Ace	18.13	−2.1	NA	−0.78	Angiotensin-converting enzyme	Enterocyte/peptidase
Hacl1	17.02	−1.22	NA	−0.62	2-hydroxyacyl-CoA lyase 1	- -/enzyme
Pck1	15.25	−1.89	NA	−1.28	Phosphoenolpyruvate carboxykinase, cytosolic [GTP]	Goblet/kinase
Smim24	9.42	−0.92	NA	−0.58^a^	Small integral membrane protein 24	-/-
Slc2a2	5.99	−2.31	−1.8	−0.54	Solute carrier family 2, facilitated glucose transporter member 2	Enterocyte/transporter
Fabp1	5.22	−1.32	−1.02	NA	Fatty acid-binding protein, liver	Enterocyte/transporter
Slc5a1	5.14	−1.23	−1.14	−0.5	Sodium/glucose cotransporter 1	Enterocyte/transporter
Fmo5	4.77	−1.02	−1.19	−0.75	Dimethylaniline monooxygenase [N-oxide-forming] 5	Enterocyte/enzyme
Maob	4.6	−1.23	NA	−0.52	Amine oxidase [flavin-containing] B	Enterocyte/enzyme
Fabp2	4.54	−1.31	−1.34	NA	Fatty acid-binding protein, intestinal	-/transporter
Sult1b1	4.52	−1.76	−2.1	−0.59	Sulfotransferase family cytosolic 1B member 1	Enterocyte/enzyme
Aldob	4.42	−1.22	−1.36	−0.84	Fructose-bisphosphate aldolase B	Enterocyte/enzyme
Apoa4	4.23	−1.41	−1.44	−0.61	Apolipoprotein A-IV	Enterocyte/transporter
Apob	3.91	−0.89	NA	−0.88	Apolipoprotein B-100	Enterocyte/transporter
Tkfc	3.48	−1.06	−0.84	NA	Triokinase/FMN cyclase	Enterocyte/kinase
Cmbl	3.13	−0.98	−0.81	−0.50^a^	Carboxymethylenebutenolidase homolog	Enterocyte/enzyme
Ugt2a3	2.85	−1.44	−1.17	−0.62	UDP-glucuronosyltransferase 2A3	Enterocyte/enzyme
Hmgcs2	2.81	−3.7	−2.95	−0.59	Hydroxymethylglutaryl-CoA synthase, mitochondrial	Stem cell/enzyme
Anxa13	2.54	−1.23	−1.19	−0.79^a^	Annexin A13	-/-
Sord	2.3	−1.86	NA	−0.87	Sorbitol dehydrogenase	-/enzyme
Iap	2.3	−0.98	NA	NA	Intestinal-type alkaline phosphatase	Enterocyte/phosphatase
Hadh	2.17	−0.71	NA	NA	Hydroxyacyl-coenzyme A dehydrogenase, mitochondrial	Enterocyte/enzyme
Acox1	1.97	−0.95	NA	−0.52	Peroxisomal acyl-coenzyme A oxidase 1	Enterocyte/enzyme
Acsl5	1.82	−1	−1.03	−0.55^a^	Long-chain-fatty-acid--CoA ligase 5	Enterocyte/enzyme
Agr2	1.57	NA	NA	0.78	Anterior gradient protein 2 homolog	Goblet/-
Acadm	1.51	−1.53	−0.86	−0.66	Medium-chain specific acyl-CoA dehydrogenase, mitochondrial	Enterocyte/enzyme

a Indicates differences that were also detected from the GFD vs. DC comparison for the RNA-seq data. The table represents a subset selected to indicate the larger changes and defined with the expression change cut-offs of an absolute difference of log_2_ intensities of 0.6 for the biopsies and 1.0 for the organoid, respectively (all with FDRs <0.05).

**TABLE 3:** Proteins associated with the ENRI cells with human analogs only detected as downregulated in the RNA-seq biopsy comparisons.

Mouse gene	Cell ENRI- ENRC (log2)	RNA-seq difference (log2)	Protein name	Marker and protein type
Prkg2	1.42	−1.02	cGMP-dependent protein kinase 2	-/kinase
Cideb	2.24	−0.98^a^	Cell death activator CIDE-B	Enterocyte/-
G6pc	15.37	−0.94	Glucose-6-phosphatase	-/phosphatase
Vnn1	1.95	−0.87	Pantetheinase	-/enzyme
Asah2	7.66	−0.81	Neutral ceramidase	-/enzyme
Slc15a1	17.55	−0.78	Solute carrier family 15 member 1	Enterocyte/transporter
Ms4a10	17.56	−0.75	Membrane-spanning 4-domains subfamily A member 10	Enterocyte/-
Pfkfb4	4.18	−0.73	6-phosphofructo-2-kinase/fructose-2,6-bisphosphatase 4	Enterocyte/kinase
Abcg5	5.19	−0.71^a^	ATP-binding cassette sub-family G member 5 (EC 7.6.2.-) (Sterolin-1)	Enterocyte/transporter
Abcc2	3.82	−0.70	Canalicular multispecific organic anion transporter 1	Enterocyte/transporter
Phyh	1.31	−0.70^a^	Phytanoyl-CoA dioxygenase, peroxisomal	-/enzyme
Cd36	17.81	−0.69	Platelet glycoprotein 4	Enterocyte/transmembrane receptor
Hpgd	16.63	−0.68^a^	15-hydroxyprostaglandin dehydrogenase [NAD (+)]	Enterocyte/enzyme
Ace2	10.28	−0.67^¥^	Angiotensin-converting enzyme 2	Enterocyte/peptidase
Galnt6	1.29	−0.65^a^	Polypeptide N-acetylgalactosaminyltransferase 6	goblet/enzyme
Enpp3	16.49	−0.64	Ectonucleotide pyrophosphatase/phosphodiesterase family member 3	-/enzyme
Cdhr2	2.74	−0.63^a^	Cadherin-related family member 2	-/-
Retsat	2.79	−0.63^a^	All-trans-retinol 13,14-reductase	Enterocyte/enzyme
Mme	7.12	−0.62	Neprilysin	Enterocyte/peptidase
Anpep	3.48	−0.62^a^	Aminopeptidase N	Enterocyte_peptidase
Tmc5	16.61	−0.60^a^	Transmembrane channel-like protein 5	-/-
Sdcbp2	3.10	−0.60^a^	Syntenin-2 (Syndecan-binding protein 2)	-/-

All figures are differences of log2 values.

^a^ Indicates differences detected from the GFD vs. DC comparison for the RNA-seq data, which notably accounted for half of the ENRI-like proteins detected in the RNA-seq data only. The table represents a subset selected to indicate the larger changes and defined with the expression change cut-offs of an absolute difference of log2 intensities of 0.6 for the biopsies and 1.0 for the organoid, respectively (all with FDRs <0.05).

In contrast to the pattern of decreased enterocyte associated proteins, polymeric immunoglobulin receptor (Pigr), vimentin (Vim), and cathepsin S (Cttss) were more abundant in the ENRI cells and upregulated in the biopsy samples (Dotsenko et al., 2020; Stamnaes et al., 2021). In the CD biopsy-derived organoid analyses by Deiterich, Vim was similarly more abundant. Additionally, NLRP6 and CCL25 were detected in the panel of innate immunity genes that were increased in the CD organoids (Freire et al., 2019). The latter two were more abundant in the ENRI cells of the mouse organoid. In contrast, pyruvate dehydrogenase kinase isoform 4 (Pdk3), Fos-related antigen 2 (Fosl2), and protein NDRG1 were associated with the ENRC cells and lower abundance in the biopsy data.

### 3.3 Comparison with other organoid and intestine proteomics data

Further evaluation of the data from this model was drawn from comparisons with other studies of the mini-gut and intestine. Notably, in this respect, Lindeboom *et al.* previously extensively characterized the minigut Lindeboom et al. (2018). The two proteomics data sets were compared to establish the similarities and differences. For the 4,500 overlapping proteins, the equivalent expression differences of the datasets were positively correlated (Supplementary Figures S4, R^2^ = 0.45).

Despite the comprehensive analysis provided by the study conducted by Lindeboom et al. (2018), assessment of the common proteins mapped to reviewed entries in the UniProt database revealed an additional 78 proteins unique to our data set (Supplementary Table S5). Out of these, 25 were differentially abundant between the two cell states (FDR ≤0.05, FC ≥ 2), with 11 more abundant in ENRI cells and 14 more abundant in the ENRC cells. Those more prominently detected in ENRI conditions included ferritin light chain 1 (Ftl1, 15 unique peptides) and calcium-activated chloride channel regulator 4A (Clca4a, 13 unique peptides). There were several proteins from large families, such as calcium-activated chloride channel regulators (Clca2 and Clca4a), cytochrome enzymes and associated reductases and oxidases, NADH dehydrogenase and oxidoreductases, and histones. In addition, there were some Paneth cell-associated defensin family members (Defa5 and Defa24), further representing the armory of antimicrobial proteins associated with the gut, and the Tuft cell markers brain acid soluble protein 1 (Basp1) and Cystm1 (cysteine-rich and transmembrane domain-containing protein 1). Other proteins that were specifically detected in these data include Mt-2 and Tp53rk.

As indicated above, the comparison with human organoids provided an important metric for our model. Freire and coworkers observed the increase in stem cell markers and decreased levels of genes associated with the gut barrier in the CD organoids. The latter included members of the mucin and claudin families, which were ENRI-associated in the mouse mini-gut model.

## 4 Discussion

*The mini-gut cellular proteome*: to create an intestinal model for CD research, a mouse mini-gut cell system was established. The protein expression profiles of the crypt- and differentiated, villi-like cells were determined using mass spectrometry-based label-free proteomics. Evaluation of protein expression changes revealed a wide repertoire of intestinal cell markers (Figure 4). In keeping with the high enterocyte composition of the villus proteome, the most numerous type of cell markers detected was for enterocytes (van der Flier and Clevers, 2009).

*Biopsy comparisons with the cellular proteome*: with the comparison of biopsy markers in published literature and our cellular proteomics data, the overlap was viewed in terms of the intestinal markers (Figures 4A–C), the ENRC and ENRI associated proteins (Figure 4D), and the changes of their analogs in the biopsy comparisons for gluten challenge. On the whole, the intestinal markers of the crypt-like state and generally the ENRC-like proteins were mostly found in the upregulated proteins/genes (165 of 181), whereas the enterocyte markers and ENRI-like proteins were downregulated (191 of 221, Figure 4D). The division is indicative of a disruption of the differentiation process and the atrophy of the intestinal villi that occurs with CD.

The DNA replication licensing factor MCM complex members (MCM2–7) were significantly more abundant in the ENRC cells and were upregulated in the proteomics biopsy after dietary challenge (Table 1). MCM5 and MCM4 were also reported in the RNA-seq biopsy data. The MCM complex is involved with the initiation of DNA replication. Notably, in the intestine, the MCMs are expressed in the crypt cells and have been shown in Lgr5+ intestinal stem cells to influence the cell cycle and proliferative fate decisions (Carroll et al., 2018). Furthermore, MCMs are overexpressed in multiple cancers (Das et al., 2014). In the context of CD, the differential expression could thus reflect a reduction in development of the mature cells.

Angiotensin-converting enzyme (Ace) is one among the prominently ENRI cell-associated protein analogs that were downregulated with gluten challenge. Although ubiquitously expressed, high levels of Ace are found in the intestine, in particular as a brush border enzyme (Bruneval et al., 1986). Likewise, the brush border enzyme trehalase was highly upregulated in the ENRI cells and downregulated in the gluten challenge data (Table 2). Other similar examples include intestinal-type alkaline phosphatase (Iap) and intestinal fatty acid-binding protein (Fabp2 and I-FABP). The latter is an important marker that is used to estimate enterocyte damage (Pelsers et al., 2005).

With the biopsy data, there were several markers where the analogs did not follow the ENRC-up/ENRI-down trend. For example, polymeric immunoglobulin receptor (PIGR/Pigr) was upregulated in the gluten challenge, and yet it was highly abundant in the ENRI cells. Pigr is involved in transcytosis of polymeric IgA and IgM across epithelia, whereupon the immunoglobulins are released and form an immunological barrier (Sano et al., 2007). In view of the central role of IgA in gut homeostasis, PIGR deficiency and disruption of transcytosis have been linked to inflammatory diseases in the gut (Kaetzel, 2014). In contrast, its elevated level has been associated with a range of different types of cancer (Ohkuma et al., 2020). Similarly, cathepsin S, differentially expressed in the gluten challenge and ENRI-organoid associated, is important for the antigen loading and subsequent function of MHC II molecules (Wilkinson et al., 2015) and has a role in pain and itching, including gastrointestinal pain.

Fos-related antigen 2 (Fosl2) and protein NDRG1 are markers with analogs associated with the ENRC cells and lower abundance in the biopsy data. Fosl2 is associated with cell proliferation and differentiation, and similarly, NDRG1 is associated with cell growth and differentiation. The latter two further indicate the challenges in maintaining the integrity of the crypt–villus axis.

Previous comparisons of quantitative proteomics and RNA-seq data have indicated how the two measures can differ (Payne, 2015; Edfors et al., 2016). Moreover, with the current evaluations, further differences concerning FFPE tissue analyses and species (i.e., human vs. mouse organoid) are likely. For the comparison of the differentially abundant ENRI and ENRC proteins, there were 78 and 278 matches that were RNA-seq or proteomics only, respectively, indicating a better overlap with the proteomics data. Nevertheless, evaluation of their nature reflected the anticipated disruptions, including the lower expression of gut markers and solute carriers and increased expression of transcriptional machinery associated with the crypt cells. Along with the use of different methodologies, i.e., RNA-seq vs. proteomics, other challenges could have arisen with analyte (protein vs. RNA) recovery, sensitivity, and potentially the numbers of individuals studied. Notably, our use of the mouse model is limited by the known dissimilarities between mice and humans (Lin et al., 2014), which could thereby reflect the level of overlapping characteristics in these comparisons.

In addition, the panel of protein markers from the study by Dotsenko and co-workers included the comparison of diseased controls (DC) vs. gluten-free diet, revealing that underlying pathologies of CD exist even with disease management (Dotsenko et al., 2020). The comparison of their data with the organoid further illustrates the expression of these makers in the model (Tables 1–3). Of the matched ENRI-associated proteins found only in the RNA-seq comparison (Table 3), half of them were distinguished when GFD was compared to DC.

Further appraisal of the detected proteome was provided by comparison with the comprehensive proteomics dataset of Lindeboom et al. (2018). On the whole, in addition to replicating many of the observations from the latter study, this also highlighted several proteins unique to our data set, as well as other aspects of the data (Supplementary Table S5). For example, Cip2a is an important oncoprotein, which was expressed preferentially in the crypt-like cells in both data sets (Lindeboom et al., 2018). Reference to our earlier GRO-seq analysis of this system reiterated the specific expression of Cip2a in the crypt-like cells (Oittinen et al., 2017), and likewise, Gebert et al. reported Cip2a detection in the mouse intestinal stem cells only (Gebert et al., 2020). Previous studies have focused on the interaction of Cip2a with Myc, including studies of the mouse intestine. In the latter, Myc expression was shown to be essential for intestinal crypt regeneration, although it is dispensable for normal tissue function and good health of the mouse. In keeping with this, GO annotation analysis of the proteins specific to the crypt-like cells highlighted associations with the cell cycle. Of the other proteins specifically detected in our data, there were several that have been related to intestinal processes and diseases. For example, metallothionein-2 (MT-2) has been associated through its anti-apoptotic and immuno-modulating effects with IBD (Waeytens et al., 2009). The Tuft cell marker Basp1 is a membrane-associated protein with several PEST motifs. PEST sequences are associated with proteins with signaling for degradation and short intra-cellular half-lives (Rogers et al., 1986), the latter being in keeping with the high turnover of cells in the intestinal lining. The differences in our results compared to those in the previous analyses may arise from protein grouping (particular for large protein families) and the database used, as well as differences in cell cultures and processing.

In summary, these analyses provide comparison of the cellular proteomes of the differentiated and un-differentiated mouse mini-gut organoid model with marker panels from human CD biopsy analyses. Although research with human organoids would be optimal (Freire et al., 2019; Dieterich et al., 2020; Dotsenko et al., 2023), these analyses demonstrate how observations from the mini-gut model are relevant to the challenges of intestinal homeostasis associated with CD. In addition to serving as an accessible and flexible disease model, further applications of the mini-gut could be in the development of new treatments and drug screens.

## Data Availability

The datasets presented in this study can be found in online repositories. The names of the repository/repositories and accession number(s) can be found below: https://www.ebi.ac.uk/pride/archive/projects/PXD025690.
