# Supplementary material for: Comparative proteomics analysis of the mouse mini-gut organoid: insights into markers of gluten challenge from celiac disease intestinal biopsies
Source: Front Mol Biosci. 2024 Aug 28;11:1446822. doi: 10.3389/fmolb.2024.1446822 (PMC11387180; doi:10.3389/fmolb.2024.1446822)
Supplement: Supplementary file 1 [file DataSheet1.DOCX]

Comparative Proteomics Analysis of the Mouse Minigut Organoid and Insights into Markers of Gluten Challenge from Celiac Disease Intestinal Biopsies

Robert Moulder^1^, Santosh Bhosale^1^, Keijo Viiri^2^, Riitta Lahesmaa^1^

Table of Contents

[**Organoid Morphology** 3](#_Toc173171687)

[**Cellular Proteomics:** 3](#_Toc173171688)

[**Figure S1**: a) A volcano plot of the cellular proteomics data of the mouse minigut 4](#_Toc173171689)

[• **Gene Ontology and Protein-protein interaction analysis** 4](#_Toc173171690)

[• **Intestinal Cell Markers** 5](#_Toc173171691)

[**Table S2:** Number of Intestinal Cell markers detected in the cellular proteome 5](#_Toc173171692)

[• **ENRI and ENRC Specific Proteins**: 5](#_Toc173171693)

[**Figure S3**: String Protein-Protein Interaction analysis of Proteins Unique to the ENRC cells 6](#_Toc173171694)

[**Table S3:** Proteins Unique to ENRC Cells 6](#_Toc173171695)

[**Table S4:** Proteins Unique to ENRI Cells 7](#_Toc173171696)

[**Data Comparisons** 8](#_Toc173171697)

[**Organoid Proteome**: 8](#_Toc173171698)

[**Figure S4:** Correlation of the relative expression changes for the differentiated cells vs. undifferentiated cells and the equivalent comparison the Lindeboom data. 8](#_Toc173171699)

[**Table S5:** Specific Proteins to the current cellular Proteome 9](#_Toc173171700)

[**Figure S5:** Proteins Specific to these data 12](#_Toc173171701)

[**Data Availability**: 13](#_Toc173171702)

[**References** 13](#_Toc173171703)

In addition to these data, the **supplementary Excel file** includes the following:

| **Sheet** | **Title** | **Content** |
| --- | --- | --- |
| 1 | Cellular_proteome: | From the MaxQuant "proteingroup.txt" file the Perseus processed and ROTS analyzed cellular data is listed |
| 2 | [Cell_Markers](file:///D:\HP005_D\Seafile\Files_I%20_might_need\MiniGut\Frontiers\Revise\Excel_supplement_Minigut_revise2.xlsx#Cell_Markers!A1) | Detected intestinal cell markers are listed together with their LFQ data as in **Figure 4** |
| 3 | RT-PCR of Markers | qRT-PCR was made for the intestinal markers for one of the organoid cultures |
| 4 | 4-day RT-PCR optimisation | qRT-PCR was made for the intestinal markers for a 4 day time course to select the optimal sample selection |
| 5 | [Biopsy-comparsions](file:///D:\HP005_D\Seafile\Files_I%20_might_need\MiniGut\Frontiers\Revise\Excel_supplement_Minigut_revise2.xlsx#'Biopsy-comparsions'!A1) | Comparison of the organoid proteome with data from celiac disease biopsy samples (RNA-seq and proteomics). Examples from these are shown in Tables 1-4. |
| 6 | RNA-seq Biopsy | Data from Dotsenko, V. et al., including GFD vs PGC and GFD vs DC. These data are used in Tables 1-4. |
| 7 | Proteomics biopsy | Data from Stamnaes, J. et al. , GFD vs PGC. These data are used in Tables 1-4. |
| 8 | MG to human | For comparing data mouse and human genes were mapped and used in the marker comparisons that are represented in Tables 1-4. |
| 9 | [Lindeboom_ROTS](file:///D:\HP005_D\Seafile\Files_I%20_might_need\MiniGut\Frontiers\Revise\Excel_supplement_Minigut_revise2.xlsx#lindeboom_ROTS!A1) | ROTS results from the analysis of the Lindeboom supplementary data are provided. The detected changes were compared and are discussed in the results section |
| 10 | [Specific Detections](file:///D:\HP005_D\Seafile\Files_I%20_might_need\MiniGut\Frontiers\Revise\Excel_supplement_Minigut_revise2.xlsx#Specific!A1) | The proteins that were not mapped to the Lindeboom et al. data are listed. The comparison of the two data sets is considered in the discussion. |

# **Organoid Morphology**

- Brightfield microscopy was used to visualize the morphology or the minigut organoids. Imaged of the ENRC and ENRI cells are shown below.


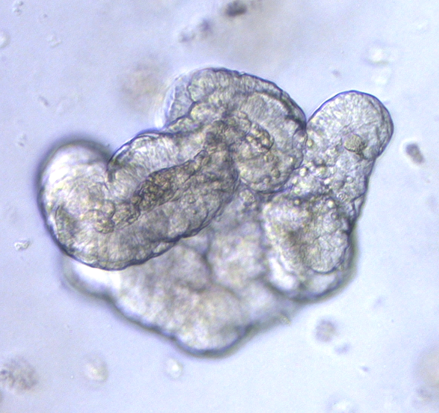

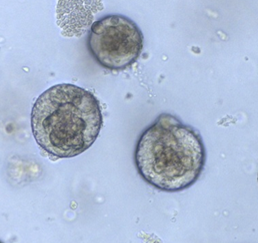


ENRC

ENRI

# **Cellular Proteomics:**

- LC-MS/MS proteomics analysis was made of a mouse minigut organoid model.
- The LC-MS/MS data was searched and quantified using MaxQuant^1,2^ and the data subsequently pre-processed with Perseus software^3^ .
- Illustrative of the contrasting cellular expression differences, a volcano plot of the cellular proteomics data is shown in **Figure S1** with accompanying hierarchical clustering of the differentially abundant proteins.


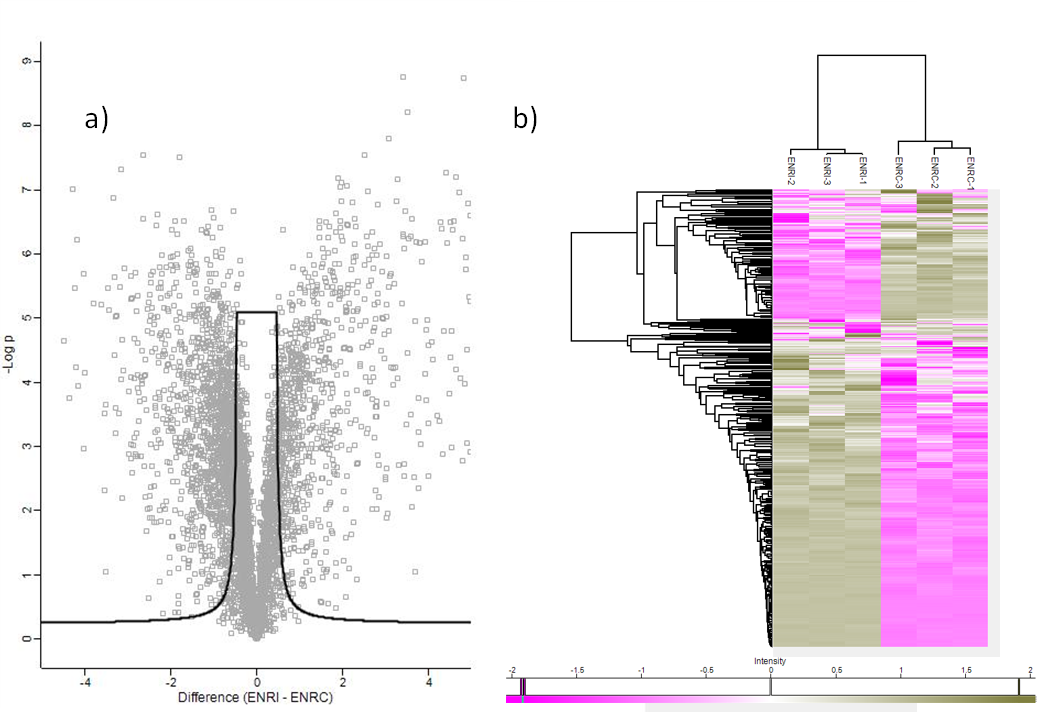


**Figure S1**: a) A volcano plot of the cellular proteomics data of the mouse minigut. Differences in log2 normalized expression vs false discovery rate (paired t-test). b) Heatmap of differentially abundant proteins after hierarchical clustering (Z-score normalized intensities). The brown and pink shaded scales indicate proteins more abundant and less abundantly, respectively.

- **Gene Ontology and Protein-protein interaction analysis** of the differentially abundant proteins was made using DAVID (<https://david.ncifcrf.gov/>) and STRING (<https://string-db.org/>). These results are shown in the Figures of the main manuscript. Notably, for the predominant pathways detected in the ENRC proteome was the reoccurring presence of series of DNA polymerases and replication factors. The STRING interaction map for these alone is indicated in **Figure S2.**


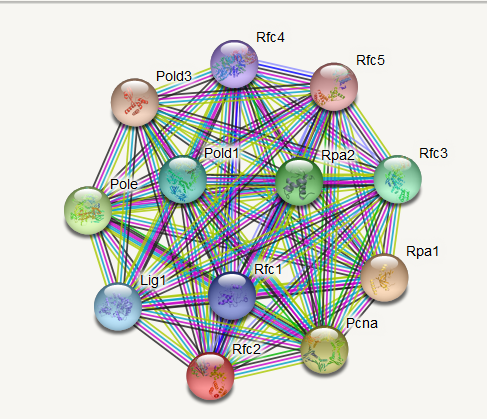
**Table S1:** DNA polymerases and replication factors enriched in the ENRC Cells

| Entry | Gene names |
| --- | --- |
| Q9EQ28 | Pold3 |
| P52431 | Pold1 |
| P17918 | Pcna |
| Q9WVF7 | Pole Pole1 |
| P37913 | Lig1 Lig-1 |
| Q9WUK4 | Rfc2 |
| Q9D0F6 | Rfc5 |
| Q99J62 | Rfc4 |
| Q8VEE4 | Rpa1 |
| Q8R323 | Rfc3 |
| Q62193 | Rpa2 Rpa34 |
| P35601 | Rfc1 Ibf-1 Recc1 |

**Figure S2:** DNA polymerases and replication factors were enriched in the ENRC Cells

- **Intestinal Cell Markers:** As notable resources for reference, Gebert *et al.* analyzed the proteomes of intestinal crypts from mice across different anatomical regions and ages^6^, and Haber and coworkers^7^ created a list of the marker genes from single cell analysis of mouse small intestine and organoids. Comparison with the Haber *et al*.^7^ lists illustrated the detection of a diversity of markers of the intestinal cell types, as summarized in **Table S1** and **Figure 4**. Further representation of the associated expression values are provide in the supplementary Excel file detailed below.

### **Table S2:** Number of Intestinal Cell markers detected in the cellular proteome

| Cell type | Markers detected | Expression specificity (villi/crypt) |
| --- | --- | --- |
| Goblet | 198 | both |
| Enterocyte | 286 | villi |
| Stem | 43 | crypt |
| Tuft | 128 | villi |
| Enteroendocrine | 16 | both |
| Paneth | 16 | crypt |

Additional representation of these data are in the supplementary Excel file.

- **ENRI and ENRC Specific Proteins**: Analysis of the cellular proteomics data revealed that some of the proteins were not detected in both cell types. Protein-protein interactions between the protein specific to the ENRC cells are shown **Figure S3**, including the oncoprotein Kiaa1524 (i.e. Cip2a). The lists of proteins specific to the cell states are shown in **Table S3** & **Table S4**.

******Figure S3**: String Protein-Protein Interaction analysis of Proteins Unique to the ENRC cells. The proteins used in the analysis are indicated in **Table S3**. (<https://string-db.org/>)

| **Table S3:** Proteins Unique to ENRC Cells | | | |
| --- | --- | --- | --- |
| T: Protein names | Razor + unique peptides | T: Majority protein IDs | T: Gene names |
| Cell division cycle protein 27 homolog | 4 | A2A6Q5 | Cdc27 |
| Rho guanine nucleotide exchange factor 10-like protein | 3 | A2AWP8 | Arhgef10l |
| Zinc finger CCHC domain-containing protein 7 | 2 | B1AX39 | Zcchc7 |
| Zinc finger CCCH domain-containing protein 13 | 3 | E9Q784 | Zc3h13 |
| Serine/threonine-protein kinase Chk1 | 2 | O35280 | Chek1 |
| Zinc finger matrin-type protein 3 | 2 | O54836 | Zmat3 |
| Hepatocyte nuclear factor 3-alpha;Hepatocyte nuclear factor 3-gamma | 2 | P35582;P35584 | Foxa1;Foxa3 |
| Leucine-rich repeats and immunoglobulin-like domains protein 1 | 3 | P70193 | Lrig1 |
| Protein ECT2 | 5 | Q07139 | Ect2 |
| Testis-expressed sequence 30 protein | 5 | Q3TUU5 | Tex30 |
| Kinesin-like protein KIF22 | 5 | Q3V300 | Kif22 |
| RelA-associated inhibitor | 2 | Q5I1X5 | Ppp1r13l |
| PWWP domain-containing protein MUM1 | 2 | Q6DID5 | Mum1 |
| Centrosomal protein of 170 kDa protein B | 3 | Q80U49 | Cep170b |
| Kinesin-like protein KIF20B | 3 | Q80WE4 | Kif20b |
| Synaptoporin | 2 | Q8BGN8 | Synpr |
| Homeobox-containing protein 1 | 2 | Q8BJA3 | Hmbox1 |
| S-adenosyl-L-methionine-dependent tRNA 4-demethylwyosine synthase | 2 | Q8BJM7 | Tyw1 |
| Ribosomal protein S6 kinase beta-1 | 2 | Q8BSK8 | Rps6kb1 |
| Protein CIP2A | 5 | Q8BWY9 | Kiaa1524 |
| Actin filament-associated protein 1-like 1 | 5 | Q8BZI0 | Afap1l1 |
| Protein zwilch homolog | 3 | Q8R060 | Zwilch |
| Zinc finger protein 830 | 4 | Q8R1N0 | Znf830 |
| Activating signal cointegrator 1 complex subunit 2 | 3 | Q91WR3 | Ascc2 |
| Leucine zipper putative tumor suppressor 2 | 2 | Q91YU6 | Lzts2 |
| SH3 domain-binding protein 4 | 2 | Q921I6 | Sh3bp4 |
| Tubulin beta-6 chain | 2 | Q922F4 | Tubb6 |
| Single-stranded DNA-binding protein 3 | 2 | Q9D032 | Ssbp3 |
| VPS10 domain-containing receptor SorCS2 | 2 | Q9EPR5 | Sorcs2 |
| Protein timeless homolog | 2 | Q9R1X4 | Timeless |
| RNA-binding protein with multiple splicing | 2 | Q9WVB0 | Rbpms |
| DNA polymerase epsilon catalytic subunit A | 6 | Q9WVF7 | Pole |
| Methyl-CpG-binding domain protein 1 | 2 | Q9Z2E2 | Mbd1 |

| **Table S4:** Proteins Unique to ENRI Cells | | | |
| --- | --- | --- | --- |
| T: Protein names | Razor + unique peptides | T: Majority protein IDs | T: Gene names |
| Alcohol dehydrogenase 4 | 13 | Q9QYY9 | Adh4 |
| Trehalase | 11 | Q9JLT2 | Treh |
| Angiotensin-converting enzyme | 7 | P09470 | Ace |
| Gamma-glutamyltranspeptidase 1 | 7 | Q60928 | Ggt1 |
| ATP-binding cassette sub-family D member 1 | 6 | P48410 | Abcd1 |
| Transmembrane channel-like protein 5 | 6 | Q32NZ6 | Tmc5 |
| Sodium- and chloride-dependent transporter XTRP3A | 6 | Q8VDB9;O88575 | Slc6a20a;Slc6a20b |
| Solute carrier family 15 member 1 | 6 | Q9JIP7 | Slc15a1 |
| H-2 class I histocompatibility antigen, TLA(B) alpha chain | 5 | P14432 | H2-T3 |
| Huntingtin | 5 | P42859 | Htt |
| Leucine-rich repeat-containing protein 57 | 5 | Q9D1G5 | Lrrc57 |
| ATP-binding cassette sub-family G member 8 | 5 | Q9DBM0 | Abcg8 |
| Serine/threonine-protein kinase DCLK1 | 5 | Q9JLM8 | Dclk1 |
| cGMP-inhibited 3,5-cyclic phosphodiesterase A | 5 | Q9Z0X4 | Pde3a |
| Dipeptidase 1 | 4 | P31428 | Dpep1 |
| RNA-binding protein 12B-B | 4 | Q66JV4;Q80YR9 | Rbm12b2;Rbm12b1 |
| Ectonucleotide pyrophosphatase/phosphodiesterase family member 3 | 4 | Q6DYE8 | Enpp3 |
| Leucine-rich repeat-containing protein 19 | 4 | Q8BZT5 | Lrrc19 |
| Chloride anion exchanger | 4 | Q9WVC8 | Slc26a3 |
| Regenerating islet-derived protein 3-gamma | 3 | O09049 | Reg3g |
| Serine/threonine-protein kinase tousled-like 2 | 3 | O55047;Q8C0V0 | Tlk2;Tlk1 |
| Platelet glycoprotein 4 | 3 | Q08857 | Cd36 |
| Galactose-3-O-sulfotransferase 2 | 3 | Q6XQH0 | Gal3st2 |
| Oxysterol-binding protein-related protein 6 | 3 | Q8BXR9 | Osbpl6 |
| Na(+)/H(+) exchange regulatory cofactor NHE-RF4 | 3 | Q99MJ6 | Pdzd3 |
| Probable N-acetyltransferase CML1 | 3 | Q9JIZ0;Q9JIY7;  E0CYC6;Q8CHQ9 | Cml1;Nat8;  Nat8b;Cml2 |
| Solute carrier family 2, facilitated glucose transporter member 5 | 3 | Q9WV38 | Slc2a5 |
| Zinc transporter 9 | 2 | Q5IRJ6 | Slc30a9 |
| Breast cancer anti-estrogen resistance protein 1 | 2 | Q61140 | Bcar1 |
| BTB/POZ domain-containing protein KCTD12 | 2 | Q6WVG3 | Kctd12 |
| Glycerophosphocholine phosphodiesterase GPCPD1 | 2 | Q8C0L9 | Gpcpd1 |
| DENN domain-containing protein 2D | 2 | Q91VV4 | Dennd2d |
| GTP-binding protein 8 | 2 | Q9CY28 | Gtpbp8 |
| 1,4-alpha-glucan-branching enzyme | 2 | Q9D6Y9 | Gbe1 |
| Large neutral amino acids transporter small subunit 2 | 2 | Q9QXW9 | Slc7a8 |
| Phosphoenolpyruvate carboxykinase, cytosolic [GTP] | 2 | Q9Z2V4 | Pck1 |

# **Data Comparisons**

**Organoid Proteome**: As a notable reference, Lindeboom *et al*. used a range of omics techniques to characterize the minigut^4^. With these measurements, they provided wide coverage of the cellular proteome by combining peptide fractionation and label-free proteomics. Comparisons were made between these data sets in terms of correlation of the protein abundance changes and GO enrichment analysis of the differentially abundant proteins. To enable these comparisons, conversion of the reported gene identifiers to reviewed UniProt protein accession numbers was made from the data provided in the supplementary information. Further analyses to ascertain the presence/absence of proteins in the data set, were made using the MaxQuant proteingroup.txt file, which was provided by the authors.

**Figure S4:** Correlation of the relative expression changes for the differentiated cells vs. undifferentiated cells and the equivalent comparison the Lindeboom data. (ENRI vs ENRC and ENV vs EN respectively)


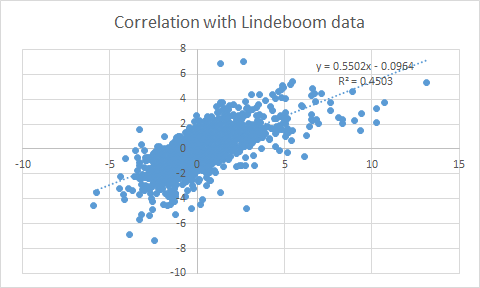


| **Table S5:** Specific Proteins to the current cellular Proteome | | | | | | |
| --- | --- | --- | --- | --- | --- | --- |
| ID | Protein names | Gene | N: Razor + unique peptides | ROTS-statistic | ROTS FDR | FC vs ENRC |
| Q6PDI5 | Proteasome adapter and scaffold protein ECM29 | Ecpas | 45 | 0.90 | 0.06 | 1.16 |
| P52482 | Ubiquitin-conjugating enzyme E2 E1 | Ube2e1 | 43 | -1.80 | 0.01 | 1.02 |
| Q3THW5 | Histone H2A.V | H2az2 | 22 | -0.47 | 0.50 | 0.53 |
| P07744 | Keratin, type II cytoskeletal 4 | Krt4 | 21 | -9.85 | 0.00 | 0.16 |
| Q9D7G0 | Ribose-phosphate pyrophosphokinase 1 | Prps1 | 15 | -2.04 | 0.00 | 0.73 |
| P29391 | Ferritin light chain 1 | Ftl1 | 15 | 2.49 | 0.00 | 2.81 |
| P02089 | Hemoglobin subunit beta-2 | Hbb-b2 | 13 | 0.74 | 0.13 | 0.81 |
| Q8K135 | Dyslexia-associated protein KIAA0319-like protein | Kiaa0319l | 13 | 7.22 | 0.00 | 3.64 |
| Q6Q473 | Calcium-activated chloride channel regulator 4A | Clca4a | 13 | 9.46 | 0.00 | 5.84 |
| P58044 | Isopentenyl-diphosphate Delta-isomerase 1 | Idi1 | 12 | -3.39 | 0.00 | 0.56 |
| Q8C7Q4 | RNA-binding protein 4 | Rbm4 | 11 | -1.04 | 0.03 | 0.39 |
| Q61286 | Transcription factor 12 | Tcf12 | 10 | -1.12 | 0.02 | 0.19 |
| O55128 | Histone deacetylase complex subunit SAP18 | Sap18 | 10 | -3.32 | 0.00 | 0.59 |
| P27661 | Histone H2AX (H2a/x) (Histone H2A.X) | H2ax | 10 | -2.40 | 0.00 | 0.64 |
| P62889 | 60S ribosomal protein L30 | Rpl30 | 9 | -3.44 | 0.00 | 0.57 |
| Q8K023 | Aldo-keto reductase family 1 member C18 | Akr1c18 | 9 | 3.84 | 0.00 | 1.92 |
| P84228 | Histone H3.2 | H3c2 | 8 | -2.26 | 0.00 | 0.72 |
| Q8BPB0 | MOB kinase activator 1B | Mob1b | 8 | -0.98 | 0.04 | 0.90 |
| Q91XV3 | Brain acid soluble protein 1 | Basp1 | 7 | -4.15 | 0.00 | 0.09 |
| P00405 | Cytochrome c oxidase subunit 2 | Mtco2 | 7 | -1.08 | 0.03 | 0.81 |
| P03975 | IgE-binding protein | Iap | 6 | -3.37 | 0.00 | 0.48 |
| O55047 | Serine/threonine-protein kinase tousled-like 2 | Tlk2 | 6 | 29.69 | 0.00 | 0.99 |
| Q9D6K5 | Synaptojanin-2-binding protein | Synj2bp | 6 | 3.43 | 0.00 | 2.02 |
| P10404 | MLV-related proviral Env polyprotein | - | 5 | -5.75 | 0.00 | 0.11 |
| O35640 | Annexin A8 | Anxa8 | 5 | -2.53 | 0.00 | 0.38 |
| P63280 | SUMO-conjugating enzyme UBC9 | Ube2i | 5 | -1.58 | 0.01 | 0.71 |
| Q9JIZ0 | Probable N-acetyltransferase CML1 | Cml1 | 5 | 36.51 | 0.00 | 0.79 |
| Q9D1L0 | Coiled-coil-helix-coiled-coil-helix domain-containing protein 2 | Chchd2 | 4 | -4.39 | 0.00 | 0.51 |
| P51807 | Dynein light chain Tctex-type 1 | Dynlt1 | 4 | 0.78 | 0.10 | 1.11 |
| Q9CQD4 | Charged multivesicular body protein 1b-2 | Chmp1b2 | 4 | 0.93 | 0.05 | 1.15 |
| Q8BHG2 | CXXC motif containing zinc binding protein | Czib | 4 | 0.85 | 0.08 | 1.21 |
| Q3UYH7 | Beta-adrenergic receptor kinase 2 | Adrbk2 | 4 | -1.03 | 0.03 | 1.46 |
| P03911 | NADH-ubiquinone oxidoreductase chain 4 | Mtnd4 | 4 | 1.29 | 0.02 | 1.49 |
| P02798 | Metallothionein-2 | Mt2 | 4 | 8.82 | 0.00 | 22.47 |
| P70269 | Cathepsin E | Ctse | 3 | -2.52 | 0.00 | 0.20 |
| Q8R1M2 | Histone H2A.J (H2a/j) | H2aj | 3 | -2.07 | 0.00 | 0.63 |
| A2AIV2 | Protein virilizer homolog | Virma | 3 | -2.10 | 0.00 | 0.64 |
| Q8K209 | Adhesion G-protein coupled receptor G1 | Adgrg1 | 3 | -1.16 | 0.02 | 0.82 |
| P00397 | Cytochrome c oxidase subunit 1 | Mtco1 | 3 | -0.06 | 0.96 | 1.03 |
| Q148V7 | RAB11-binding protein RELCH | Relch | 3 | 0.71 | 0.14 | 1.13 |
| P03930 | ATP synthase protein 8 | Mtatp8 | 3 | 1.16 | 0.02 | 1.19 |
| P03921 | NADH-ubiquinone oxidoreductase chain 5 | Mtnd5 | 3 | 1.43 | 0.01 | 1.26 |
| Q91ZR3 | CREB/ATF bZIP transcription factor | Crebzf | 3 | -1.77 | 0.01 | 1.55 |
| Q69Z23 | Dynein heavy chain 17, axonemal | Dnah17 | 3 | 0.33 | 0.70 | 1.57 |
| P29595 | NEDD8 (Neddylin) | Nedd8 | 2 | -1.11 | 0.02 | 0.11 |
| Q5G865 | Alpha-defensin 24 | Defa24 | 2 | -3.08 | 0.00 | 0.14 |
| Q9D173 | Mitochondrial import receptor subunit TOM7 homolog | Tomm7 | 2 | -3.40 | 0.00 | 0.27 |
| Q6ZWY9 | Histone H2B type 1-C/E/G | H2bc4 | 2 | -1.38 | 0.01 | 0.35 |
| Q9D2J4 | V-set and immunoglobulin domain-containing protein 1 | Vsig1 | 2 | -2.36 | 0.00 | 0.35 |
| Q925G2 | Cytochrome b reductase 1 (EC 1.-.-.-) (Duodenal cytochrome b) | Cybrd1 | 2 | -2.16 | 0.00 | 0.50 |
| Q9JLC8 | Sacsin | Sacs | 2 | -1.50 | 0.01 | 0.52 |
| O35216 | Histone H3-like centromeric protein A | Cenpa | 2 | -2.58 | 0.00 | 0.56 |
| P28312 | Alpha-defensin 5 | Defa5 | 2 | -3.35 | 0.00 | 0.57 |
| Q3U6N9 | UPF0488 protein C8orf33 homolog |  | 2 | -2.07 | 0.00 | 0.63 |
| P50714 | Alpha-defensin 16 (Defensin-related cryptdin-16) | Defa16 | 2 | -4.65 | 0.00 | 0.64 |
| P17533 | Alpha-defensin-related sequence 1 | Defa-rs1 | 2 | -1.86 | 0.01 | 0.69 |
| Q45VN2 | Alpha-defensin 20 | Defa20 | 2 | -0.81 | 0.09 | 0.76 |
| Q6PKN7 | Protein INCA1 | Inca1 | 2 | -1.19 | 0.02 | 0.76 |
| Q8R003 | Muscleblind-like protein 3 | Mbnl3 | 2 | -1.21 | 0.02 | 0.76 |
| Q99PG4 | Regulator of G-protein signaling 18 (RGS18) | Rgs18 | 2 | -1.16 | 0.02 | 0.82 |
| Q8K353 | Cysteine-rich and transmembrane domain-containing protein 1 | Cystm1 | 2 | -0.75 | 0.12 | 0.85 |
| A2AGX3 | PR domain-containing protein 11 (EC 2.1.1.-) | Prdm11 | 2 | -0.37 | 0.66 | 0.91 |
| Q5SS00 | DBF4-type zinc finger-containing protein 2 homolog | Zdbf2 | 2 | -0.98 | 0.04 | 0.93 |
| Q61468 | Mesothelin | Msln | 2 | 1.00 | 0.04 | 1.12 |
| Q9CZ69 | CKLF-like MARVEL transmembrane domain-containing protein 6 | Cmtm6 | 2 | 0.99 | 0.04 | 1.15 |
| Q8BG22 | Calcium-activated chloride channel regulator 2 | Clca2 | 2 | 0.72 | 0.13 | 1.28 |
| Q80W93 | Hydrocephalus-inducing protein (Protein Hy-3) | Hydin | 2 | 1.38 | 0.01 | 1.40 |
| F8VPN2 | Testis-expressed protein 15 | Tex15 | 2 | 1.64 | 0.01 | 1.45 |
| Q99N16 | Cytochrome P450 4F3 | Cyp4f3 | 2 | 1.95 | 0.00 | 1.49 |
| Q9DB10 | Essential MCU regulator, mitochondrial | Smdt1 | 2 | 2.24 | 0.00 | 1.66 |
| P0DN34 | NADH dehydrogenase [ubiquinone] 1 beta subcomplex subunit 1 | Ndufb1 | 2 | 3.33 | 0.00 | 1.85 |
| Q3UNB8 | Transmembrane protein 253 | Tmem253 | 2 | 2.47 | 0.00 | 2.51 |
| P09240 | Cholecystokinin | Cck | 2 | 3.81 | 0.00 | 2.77 |
| P56654 | Cytochrome P450 2C37 | Cyp2c37 | 2 | 5.29 | 0.00 | 3.08 |
| Q80XB4 | Nebulin-related-anchoring protein | Nrap | 2 | 10.67 | 0.00 | 43.19 |
| O55071 | Cytochrome P450 2B19 | Cyp2b19 | 2 | 8.70 | 0.00 | 64.33 |
| Q8BGN8 | Synaptoporin | Synpr | 2 | -54.10 | 0.00 | <0.01 |
| Q99PW4 | EKC/KEOPS complex subunit Tp53rk | Tp53rk | 2 | 1.90 | 0.00 | >100 |


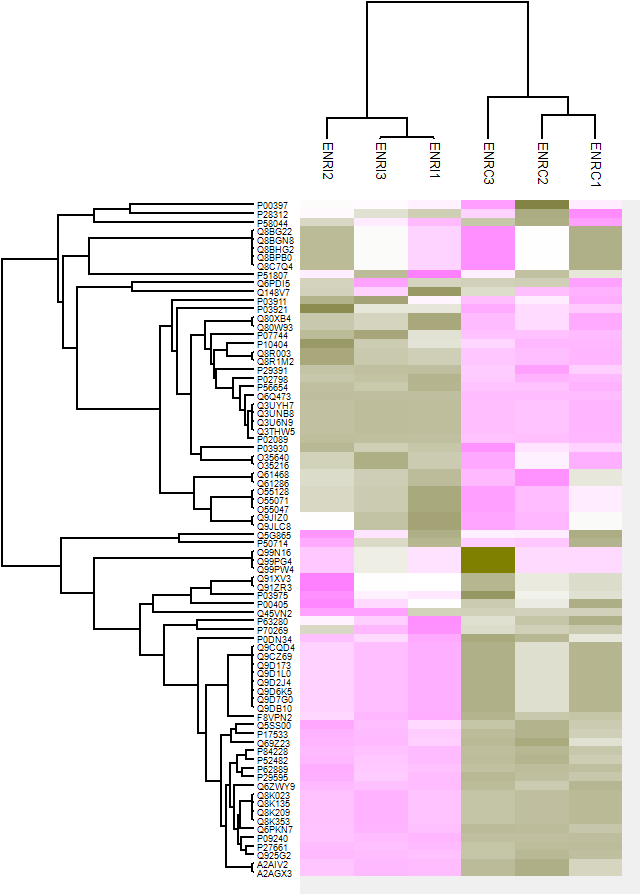


**Figure S5:** Proteins Specific to these data (not reported by Lindeboom et al.). The heatmap represents hierarchical clustering of the Z score normalized LFQ intensities.

# **Data Availability**:

These mass spectrometry proteomics data have been deposited to the ProteomeXchange Consortium (http://proteomecentral.proteomexchange.org) via the PRIDE partner repository^17^ with the dataset identifier PXD023737.

# **References**

1. Cox J, Neuhauser N, Michalski A, Scheltema RA, Olsen JV, Mann M. Andromeda: A peptide search engine integrated into the MaxQuant environment. *J Proteome Res*. 2011;10(4):1794-1805.

2. Cox J, Mann M. MaxQuant enables high peptide identification rates, individualized p.p.b.-range mass accuracies and proteome-wide protein quantification. *Nat Biotechnol*. 2008;26(12):1367-1372.

3. Tyanova S, Temu T, Sinitcyn P, et al. The perseus computational platform for comprehensive analysis of (prote)omics data. *Nat Methods*. 2016.

4. Gebert N, Cheng C, Kirkpatrick JM, et al. Region-specific proteome changes of the intestinal epithelium during aging and dietary restriction. *Cell Reports*. 2020;31(4):107565. doi: <https://doi.org/10.1016/j.celrep.2020.107565>.

5. Haber AL, Biton M, Rogel N, et al. A single-cell survey of the small intestinal epithelium. *Nature*. 2017;551(7680):333-339.

6. Lindeboom RGH, van Voorthuijsen L, Oost KC, et al. Integrative multi-omics analysis of intestinal organoid differentiation. *Mol Syst Biol*. 2018;14(6):e8227.

7. Elo LL, Hiissa J, Tuimala J, Kallio A, Korpelainen E, Aittokallio T. Optimized detection of differential expression in global profiling experiments: Case studies in clinical transcriptomic and quantitative proteomic datasets. *Brief Bioinform*. 2009;10(5):547-555.

8. Dotsenko V, Oittinen M, Taavela J, et al. Genome-wide transcriptomic analysis of intestinal mucosa in celiac disease patients on a gluten-free diet and postgluten challenge. *Cell Mol Gastroenterol Hepatol*. 2020;11(1):13-32.

9. Perez-Riverol Y, Csordas A, Bai J, et al. The PRIDE database and related tools and resources in 2019: Improving support for quantification data. *Nucleic Acids Res*. 2019;47(D1):D442-D450.
